# Development and content validation of a symptom assessment for eosinophilic gastritis and eosinophilic gastroenteritis in adults and adolescents

**DOI:** 10.1186/s13023-021-02107-6

**Published:** 2021-11-24

**Authors:** Calvin N. Ho, Sean O’Quinn, Julie Bailey, Oren Meyers, Ashley F. Slagle, Evan S. Dellon, Catherine Datto

**Affiliations:** 1 AstraZeneca, Gaithersburg, MD USA; 2 IQVIA, Real World Solutions, Patient Centered Solutions, New York, NY USA; 3 Aspen Consulting, Steamboat Springs, CO USA; 4 Center for Esophageal Diseases and Swallowing, Division of Gastroenterology and Hepatology, University of North Carolina at Chapel Hill School of Medicine, Chapel Hill, NC USA

**Keywords:** Eosinophilic gastritis, Eosinophilic gastroenteritis, Eosinophilic duodenitis, Patient reported outcomes

## Abstract

**Background:**

A patient reported outcome (PRO) instrument with evidence of validity and reliability for assessing symptoms of eosinophilic gastritis (EG) and eosinophilic gastroenteritis (EGE) is needed to measure treatment benefit in clinical trials. The aim of this research is to develop an EG/EGE symptom PRO instrument for patients aged 12 and above.

**Methods:**

The Symptom Assessment for Gastrointestinal Eosinophilic Diseases (SAGED) was developed through a literature review, discussions with expert clinicians, and concept elicitation and cognitive debriefing interviews with patients. Patients (n = 28) were recruited based on confirmed diagnosis and self-reported symptoms. The final instrument was translated and linguistically validated with additional cognitive debriefing interviews (n = 105).

**Results:**

SAGED is a 24-h recall questionnaire consisting of eight items evaluating the core symptoms of EG and EGE (abdominal pain, nausea, bloating, early satiety, loss of appetite, vomiting, and diarrhea). Seven of the eight items are evaluated on an 11-point numerical rating scale ranging from ‘none’ to ‘worst imaginable’. Cognitive debriefing interviews showed that adults and adolescents understand the content and are able to select a response that reflects their experience. The linguistic validation process produced 21 translations that are understandable to patients and conceptually equivalent to the source version.

**Conclusions:**

SAGED is suitable for measuring symptom improvement in adult and adolescent patients with EG and/or EGE. The content validity of SAGED has been established through best practices in qualitative research for PRO instrument development. The psychometric properties of SAGED will be evaluated in a future study.

## Background

Eosinophilic gastritis (EG) and eosinophilic gastroenteritis (EGE) are rare disorders characterized by chronic gastrointestinal symptoms along with pathologic eosinophilic inflammation in the stomach (EG) and/or small intestine (EGE) in the absence of secondary causes of eosinophilia. EG and EGE, along with eosinophilic esophagitis (EoE) and eosinophilic colitis (EC), fall under the umbrella of eosinophilic gastrointestinal diseases (EGIDs).

In the United States, prevalence of EG has been estimated at 6.3/100,000 and EGE at 8.4/100,000 [1]. These estimates may be conservative, as patients may have been misdiagnosed with other functional gastrointestinal disorders due to their nonspecific symptoms [2, 3]. Many patients experience a long delay in receiving an EG/EGE diagnosis [4]. Clinical presentation of EG/EGE varies based on the site of inflammation, but typical symptoms reported in the literature include abdominal pain, nausea, and vomiting [5–9]. Signs include malabsorption, weight loss, mucosal ulcerations, anemia, and protein-losing enteropathy [10–14]. Additionally, patients often experience disease-related psychological, social, and financial issues [15].

The symptoms and treatments for EG/EGE can cause serious side effects and reduced quality of life. Current treatments for EG/EGE include restrictive diets and systemic corticosteroids [16–18]. Diets that eliminate trigger foods may be effective in certain patients, but results are variable, and no conclusive evidence is available regarding long-term effectiveness [19]. Though treatment with systemic corticosteroids can result in clinical improvement, long-term use of corticosteroids is limited due to harmful side effects [20]. Additionally, up to 65% of patients with EGE are not responsive or are only partially responsive to treatment, including oral steroids [4, 21].

Despite the high unmet medical need for EG and EGE patients, drug development in this area has been slow. One of the major challenges is that there is no publicly available patient reported outcome (PRO) assessment fit for the purpose of measuring EG or EGE symptoms in a clinical trial setting. Though eosinophilia in gastrointestinal tissue is necessary to make an EGID diagnosis, several studies in EoE have shown that reduction in tissue eosinophils may not correlate with reduction in symptoms [22–25]. Thus, regulators now expect a fit for purpose patient-reported symptom measure to be used as a primary endpoint in EoE trials [27]. No such instrument exists for EG/EGE.

A symptom assessment with documented content validity and acceptable psychometric properties is needed to address the needs of EG/EGE clinical studies. In the present study, we developed the Symptom Assessment for Gastrointestinal Eosinophilic Disorders (SAGED),^1^ a 24-h recall assessment of the most common symptoms of EG and EGE suitable for patients aged 12 and above. SAGED was developed according to good principles of instrument development, which supports its use as a primary endpoint in a clinical trial for EG/EGE [28–32]. This article outlines the development of SAGED based on qualitative research, including conceptual framework development, concept elicitation interviews with patients, item generation, cognitive interviewing with additional patients, and linguistic validation (Tables 1, 2, 3, 4, 5, 6).

**Table 1:** Summary demographics of 28 patient interview respondents (waves 1–6)

Demographic characteristic	Number of patients in wave 1 (n = 5)	Number of patients in wave 2 (n = 5)	Number of patients in wave 3 (n = 5)	Number of patients in wave 4 (n = 4)	Number of patients in wave 5 (n = 4)	Number of patients in wave 6 (n = 5)	Total number of patients (n = 28)
*Age (years)*							
12–17	1	1	2	1	0	1	6
18–28	1	2	3	1	1	0	8
29–39	1	2	0	1	2	2	8
40–49	0	0	0	0	1	1	2
50–64	2	0	0	1	0	1	4
*Sex*							
Male	2	2	2	2	1	2	11
Female	3	3	3	2	3	3	17
*Diagnosis*							
EG only	2	1	1	1	1	1	7
EGE only	0	1	2	0	1	1	5
EC only	0	0	0	0	2	2	4
EG and EGE	0	1	0	0	0	0	1
EGE and EC	0	2	0	0	0	0	2
EoE and EG	1	0	0	1	0	0	2
EoE and EGE	1	0	2	1	0	1	5
EoE, EG, and EGE	1	0	0	0	0	0	1
EoE, EG, EGE, and EC	0	0	0	1	0	0	1

**Table 2:** Abbreviated conceptual model of EG and EGE

Symptoms	Impacts
Gastrointestinal	Dietary
Nausea	Fear of trying new foods
Vomiting	Sadness over loss of favorite foods
Diarrhea	Being hungry (adolescents)
Bloating (abdominal distention)	Emotional
Constipation	Feeling misunderstood
Pain	Feeling hopeless or miserable for having the disease
Abdominal pain	Depression
Appetite	Stress about disease
Lack of appetite	Worry about disease
Early satiety	Frustration about disease
	Frustration about diet
	Social relationships
	Missing out on social activities

**Table 3:** Symptoms mentioned in concept elicitation interviews and their disturbance ratings

Symptom	Total patients mentioning (n = 23) (%)	Patients mentioning concept (spontaneous)	Patients mentioning concept (probed)	Average disturbance rating*
Abdominal pain	22 (96%)	22	0	8.3
Nausea	21 (91%)	16	5	7.1
Diarrhea	21 (91%)	14	7	5.4
Fatigue	19 (83%)	10	9	7.3
Vomiting	16 (70%)	16	0	5.9
Heartburn	16 (70%)	7	9	5.6
Bloating	16 (70%)	9	7	4.8
Lack of appetite	16 (70%)	5	11	4.4
Early satiety	15 (65%)	5	10	5.6
Constipation	13 (57%)	10	3	5.0
Gassiness/burping	14 (61%)	7	7	5.5
More frequent bowel movements	13 (57%)	5	8	5.8
Feeling hungry	13 (57%)	3	10	5.6
Flank pain	9 (39%)	3	6	6.5
Muscle pain	8 (35%)	2	6	5.0
Larger bowel movements	7 (30%)	3	4	5.2
Pain/discomfort in upper abdomen	7 (30%)	4	3	6.8
Acid reflux	9 (39%)	7	2	7.2
Food aversions	5 (22%)	3	2	7.4
Chest pain	6 (26%)	2	4	4.7
Joint pain	6 (26%)	1	5	7.3
Stiff fingers, toes, and neck	5 (22%)	2	3	5.9
Dry mouth	4 (17%)	2	2	3.7
Trouble swallowing	2 (9%)	2	0	7.5
Heightened sense of smell	2 (9%)	2	0	3.5
Failure to thrive/growth retardation	2 (9%)	1	1	9.5
Dehydration	2 (9%)	2	0	3.0
Disorientation	1 (4%)	1	0	6.0
Numb fingers/toes	2 (9%)	1	1	7.5
Hot flashes	2 (9%)	1	1	1.7
Food stuck in esophagus	2 (9%)	1	1	Not reported
Choking	1 (4%)	1	0	Not reported
Trouble digesting	1 (4%)	1	0	Not reported

*Disturbance ratings were assessed on a 0–10 scale, where 0 = 'not disturbing at all' and 10 =  'extremely disturbing'

**Table 4:** Symptom frequency and disturbance ratings for core symptoms

Symptom	Total patients mentioning			Average disturbance rating*		
	EG only (n = 6)	EGE only (n = 4)	EG/EGE (n = 1)	EG only (n = 6)	EGE only (n = 4)	EG/EGE (n = 1)
Abdominal pain	6 (100%)	4 (100%)	1 (100%)	8.2	8.7	10.0
Nausea	6 (100%)	4 (100%)	1 (100%)	7.6	7.2	10.0
Vomiting	3 (50%)	4 (100%)	1 (100%)	5.3	4.7	10.0
Bloating	5 (83%)	2 (50%)	0 (0%)	6.5	4.0	N/A
Early satiety	5 (83%)	1 (25%)	1 (100%)	6.0	3.0	9.0
Lack of appetite	3 (50%)	3 (75%)	1 (100%)	6.3	5.3	5.0
Diarrhea	6 (100%)	4 (100%)	1 (100%)	4.2	5.3	7.0

*Disturbance ratings were assessed on a 0–10 scale, where 0 = 'not disturbing at all' and 10 = 'extremely disturbing'

**Table 5:** Final SAGED items (English version for the United States [source version for translations])

Item Number	Item text	Response options
Q1	During the past 24 hours, how would you rate your worst abdominal pain?	11-point NRS (‘None’ to ‘Worst imaginable’)
Q2	During the past 24 hours, how would you rate your worst nausea?	11-point NRS (‘None’ to ‘Worst imaginable’)
Q3	During the past 24 hours, how would you rate your worst bloating?	11-point NRS (‘None’ to ‘Worst imaginable’)
Q4	During the past 24 hours, how would you rate your experience of feeling full quickly when eating?	11-point NRS (‘None’ to ‘Worst imaginable’)
Q5	During the past 24 hours, how would you rate your experience of loss of appetite?	11-point NRS (‘None’ to ‘Worst imaginable’)
Q6	During the past 24 hours, how would you rate your worst diarrhea?	11-point NRS (‘None’ to ‘Worst imaginable’)
Q7	During the past 24 hours, how many times did you vomit, defined as throwing up with food or liquid coming out?	Free number entry
Q8	During the past 24 hours, how would you rate your vomiting?	11-point NRS (‘None’ to ‘Worst imaginable’)

**Table 6:** Results of cognitive debriefing of SAGED translations

Language	Country	n	Age range	Range of years of education
Arabic	Israel	5	18–75	10–16
Chinese	China	5	36–65	9–16
Dutch	Netherlands	5	18–55	15–20
English	Australia	5	18–65	9–16
English	Canada	5	18–75	10–16
French	Canada	5	26–65	10–16
French	France	5	26–64	14–16
French	Switzerland	5	26–85	11–13
German	Germany	5	26–65	9–13
German	Switzerland	5	26–75	11–16
Hebrew	Israel	5	18–85	12–20
Italian	Italy	5	36–75	8–16
Italian	Switzerland	5	18–75	11–16
Japanese	Japan	5	18–75	9–17
Polish	Poland	5	36–75	11–17
Portuguese	Brazil	5	18–75	11–16
Russian	Israel	5	26–95	10–16
Spanish	Spain	5	26–65	10–15
Spanish	United States	5	18–65	10–15
Ukrainian	Ukraine	5	26–65	11–15
Vietnamese	Vietnam	5	18–55	6–16

## Methods

### Initial identification of concepts

The first steps in PRO instrument development are defining the patient population of interest, determining the concepts that are important to measure in the population, and mapping the relationship between these concepts [28–32]. The initial target population for the instrument (and for the clinical trial program for which the instrument was to be developed) was patients aged 12 and over with EG, EGE, and/or EC. A literature review and expert interviews were conducted in tandem, with the goal of identifying an existing gastrointestinal PRO instrument that could be evaluated or modified for use in the EG, EGE, or EC populations. Research and review articles published in English between 2008–2018 focusing on symptoms and health-related quality of life in adult or adolescent patients with EG, EGE, and/or EC were compiled from PubMed. A similar literature review was conducted 12 months later to determine whether assessments for diseases with similar symptomatology could be modified for the non-EoE EGID population. As no tools appropriate for measuring EGID symptoms were found in these searches, the project shifted to developing a de novo instrument.

Four US-based expert clinicians were interviewed via telephone by trained interviewers. All four gastroenterologists were recognized experts in EGIDs; saw at least five EG, EGE, or EC patients per month; and had published scientific and/or clinical articles on EG, EGE and EC. A semi-structured discussion guide was developed to seek the clinical experts’ input on the preliminary conceptual models; the PRO instruments that could be applicable for measuring the EG, EGE, and EC patient experience; and the most appropriate PRO endpoints to measure treatment benefit.

### Concept elicitation and instrument drafting

Semi-structured interviews lasting between 60 and 90 min were conducted with patients with EG, EGE, and/or EC (n = 28). Participants were recruited through patient advocacy groups and patient recruitment vendors. Outreach methods included emails to potential patients, social media and website posts, advertisements in newsletters, and paper flyers at patient-focused conferences and meetings. All study materials were reviewed and approved by the New England Institutional Review Board. All patients gave their informed consent to be included in the study.

Patients were eligible for the study if they were 12 years of age or older; had a confirmed diagnosis of EG, EGE, or EC; were currently receiving treatment for one of those conditions; experienced gastrointestinal symptoms of one of those conditions in the past four weeks; could provide informed consent or assent to participate in the study; were resident in the US or Australia; and were able to communicate proficiently in English. Participants were required to confirm their diagnosis through a record from their healthcare provider.

**Fig. 1:**
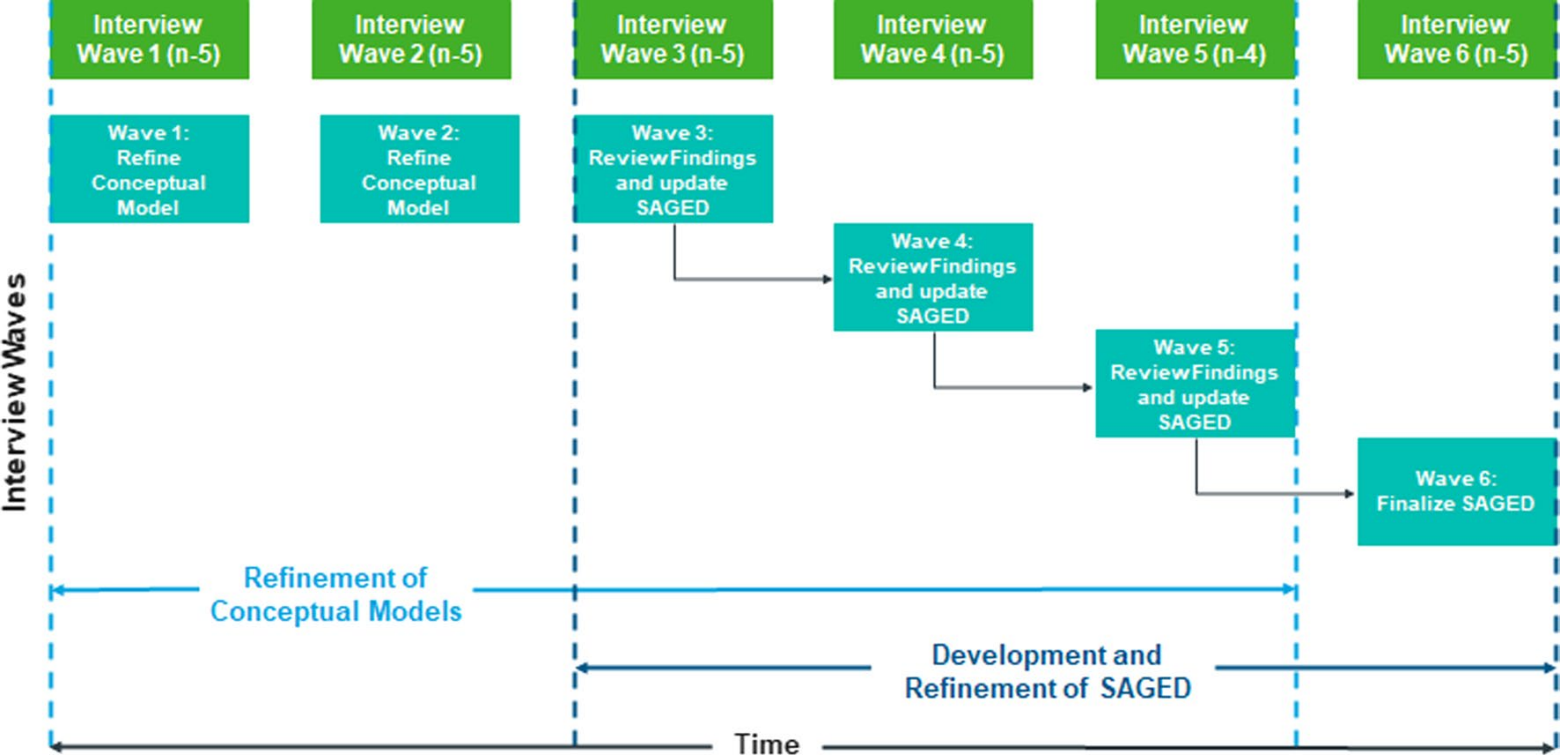
Overview of interview waves

The interviews were based on a standardized interview guide. Interviews were audio recorded, transcribed verbatim, and coded using ATLAS.ti qualitative data analysis software. As in most qualitative research in the grounded theory tradition, the sample size was not determined a priori. Recruitment was capped when the team reached theoretical saturation (the point at which additional data collection does not result in any additional concepts or refinement of existing concepts) [33, 34]. For practical reasons, the interviews were split into six waves (Fig. 1). Saturation was pre-defined as the absence of any new spontaneous concept mentions for an entire wave of concept elicitation (CE) interviews.

In CE interviews (waves 1–5), symptoms and impacts that patients spontaneously mentioned were recorded. Then, patients were probed on additional symptoms and impacts that were derived from the literature, clinician interviews, or previous patient interviews. Patients were asked to rate the bothersomeness of each symptom and impact that affected them. Between each wave of interviews, the research team reviewed the qualitative data collected so far and analyzed any newly identified symptoms or impacts. If patients spontaneously mentioned a previously unidentified symptom or impact, the study team probed on this in subsequent interviews and considered the concept for inclusion in future iterations of the conceptual model.

Following the literature review, clinician interviews and the first two waves of patient interviews (n = 10), researchers reviewed salient concepts and the language used by patients to describe these concepts in an item generation workshop. Concepts were prioritized for inclusion in the draft PRO instrument based on the frequency of spontaneous and probed mentions, average bothersomeness ratings, and the hypothesized relationship between the symptom and the known mechanisms of the disease. Items were developed for each prioritized concept based on patient language from the CE interviews and examples of other PRO items that measure similar concepts. The item generation workshop resulted in the draft SAGED that was refined in four waves of cognitive interviews.

### Cognitive interviewing, finalization of the instrument, and linguistic validation

In cognitive interviewing (CI; waves 3–6), patients were asked to complete the draft SAGED using screenshots shared via an online screen sharing platform. They were prompted to read questions aloud and respond with minimal prompting from the interviewer. Once patients had completed the full draft questionnaire, the interviewer returned to the start of the instrument and discussed each item with the patient. Patients were asked to provide feedback on questionnaire instructions, the relevance of each item, the meaningfulness of the response options, and whether there were any missing or redundant items. CI results were reviewed after each wave and adjustments to the draft instrument were made as appropriate. Adjustments were documented in an item tracking matrix. The instrument was finalized after no additional changes emerged from the last wave of cognitive interviewing.

The final SAGED was translated and linguistically validated according to the best practices outlined by the ISPOR Task Force for Translation and Cultural Adaptation [35]. Versions of SAGED were created in 15 languages for 16 countries (21 translated versions in total) (Table 6). Two independent forward translations per language were commissioned based on the source text in US English and on a concept elaboration guide developed in collaboration with the authors. The two independent translations were then reconciled into a single version. A third independent translator produced a single back translation into English for author review. Back translations were reviewed to ensure that the translation accurately represented the concepts measured in the source text.

The reconciled translation was cognitively debriefed with five native speaker patients in the target countries. The cognitive debriefing interviews determined whether the patients understood the translations in the same way that US English-speaking patients understood the source text. All notes from the interviews, including any suggested refinements, were reviewed by the authors to determine whether any changes needed to be made to the translations and/or the source text.

## Results

### Patient demographics

Twenty-eight patients were interviewed for the concept elicitation and initial cognitive debriefing stages of this study (Table 1). Twenty-two patients were adults (ages 18–64) and six were adolescents (ages 12–17). Twelve patients had a single clinician-confirmed diagnosis of EG, EGE, or EC, while the remainder had multiple EGID diagnoses. Taken together, 12 patients in the sample had an EG diagnosis, 15 had an EGE diagnosis, seven had an EC diagnosis, and nine had an EoE diagnosis. Though recruitment was open in both the US and Australia, no Australian patients were ultimately recruited.

### Concept elicitation

In the interviews with a CE component (n = 23), the most commonly reported symptoms included abdominal pain, nausea, diarrhea, and fatigue. Vomiting, heartburn, bloating, lack of appetite, early satiety, constipation, and fatigue were also commonly mentioned (Table 2). Saturation of symptoms was reached with wave 4. Salient symptoms (those symptoms with a disturbance rating > 5 and/or mentioned by at least 50% of patients in CE) were the same for patients with EG and/or EGE. Abdominal pain, nausea, vomiting, bloating, diarrhea, early satiety, and lack of appetite were confirmed to be central to the patient experience of EG and EGE regardless of age or gender.

The most common spontaneously mentioned symptom was abdominal pain. Many patients began the CE interviews with a spontaneous description of their abdominal pain. The typical location of the pain was in the lower abdomen, though some patients reported that the pain would spread:

For me, it first starts right under the ribcage where the stomach is located, or the small intestine.... It eventually gets so bad to me that it goes through my entire torso, and it starts wrapping around my lower back.

(Interview 71632464, 12 March 2019)

When probed about whether she experienced ‘chest pain’ or ‘throat pain’, this 35-year-old female EG patient said that she did not have either type. This response was common among patients who reported spreading EG- or EGE-related pain but did not have a diagnosis of EoE.

Patients reported that symptoms varied from day to day, with not all symptoms necessarily occurring together, and that symptom severity also varied day to day. This suggested a daily diary with 24-h recall approach would be the most appropriate design for a de novo symptom PRO instrument. Vomiting and diarrhea were relatively infrequent and often indicative of a severe EG/EGE episode. For instance, in a concept elicitation interview, an 18-year-old female EGE patient described how vomiting was a key feature of ‘bad’ days:

On a particularly bad day, I end up having to call into work sick. Usually, those days start the night before. I know I’m feeling sick…. I can’t sleep through the night those nights, so I’m waking up, I’m throwing up, I’m coming back to bed, sleeping for an hour, waking up, throwing up….

When asked to describe her ‘typical’ day earlier in the interview, however, the patient mostly described nausea, abdominal pain, and loss of appetite.

### Instrument development, cognitive interviewing and linguistic validation

Analysis of the concept elicitation interviews, expert clinician interviews, and literature review informed the development of the draft SAGED. All three data sources were consistent in identifying abdominal pain, nausea, bloating, early satiety, loss of appetite, vomiting, and diarrhea as core symptoms of both EG and EGE (Table 2).

In the patient interviews, heartburn and constipation were also commonly mentioned as disturbing symptoms (Table 2). However, the research team decided not to develop items for those two symptoms. Heartburn was excluded from SAGED because it is particularly associated with esophageal dysfunction, whereas the other symptoms assessed by the tool focus on symptoms related to the stomach and small intestine. The frequency of constipation as a relevant symptom was an unexpected finding for the research team; expert clinicians consulted about this suggested that it may have been a side effect of antidiarrheal medications. Fatigue was also mentioned as a disturbing symptom by many patients (Table 3). However, as fatigue is not necessarily directly related to eosinophilic inflammation in gastrointestinal tissue, it is less germane to inclusion in an instrument where all the other symptoms were gastrointestinal.

While the number of items and wording used in the draft SAGED was refined in each wave, the core symptoms covered by the instrument were confirmed as relevant by patients and the evidence did not support including any additional concepts. Seven core concepts in the initial draft SAGED (abdominal pain, nausea, bloating, early satiety, loss of appetite, vomiting, and diarrhea) were considered relevant throughout cognitive interviewing and remained in the instrument. The final version of the instrument was tested in the last wave of interviews, with no changes warranted.

The cognitive debriefing interviews from the linguistic validation process included n = 105 patients from 16 countries, speaking 15 languages. Patients had a wide range of ages and education levels (Table 6). After reviewing the results of all the linguistic validation interviews, it was determined that no changes to SAGED were indicated. Only in one set of cognitive interviews (French for Canada) did multiple patients raise the same comprehension issue. This was resolved by changing the word choice in a specific item to the locally preferred term.

## Final SAGED

The final SAGED (Table 5) is an 8-item PRO assessment that is intended to measure the severity of abdominal pain, nausea, vomiting, bloating, diarrhea, early satiety, lack of appetite, and frequency of vomiting, within the previous 24 h. Severity of worst abdominal pain, nausea, vomiting, bloating, and diarrhea are measured on a 11-point numerical rating scale (NRS), where 0 = ‘none’ and 10 = ‘worst imaginable’. The severity of experience of early satiety (termed ‘feeling full quickly’ to reflect patient language) and lack of appetite is also measured on this 11-point scale. Vomiting frequency is measured as the absolute count of vomiting during the recall period. A diagram of a torso with the abdomen circled in red accompanies the abdominal pain item.

The preliminary SAGED scoring system includes a total SAGED score composed of responses to items 1–5, and separate scores for item 6 (diarrhea severity), item 7 (vomiting count), and item 8 (vomiting severity). The total SAGED score (range: 0–50, with higher scores indicating greater symptom severity) is calculated by summing the daily responses to items 1–5 every day for 14 days and taking the mean of those daily sums. Items 6–8 are not included in the total score as vomiting is relatively infrequent. Psychometric analyses (e.g., item-to-item correlations and factor analyses) are planned to determine the most appropriate scoring algorithm for this instrument.

## Discussion

Understanding the symptoms of EG and EGE from the patient’s perspective is crucial for developing treatments for this underserved patient population. As a PRO instrument with strong evidence of content validity, SAGED is appropriate for measuring symptomatic improvement in EG/EGE clinical trials for EG/EGE. Patient interviews, literature reviews, and discussions with expert clinicians have confirmed that abdominal pain, nausea, vomiting, bloating, diarrhea, early satiety, and lack of appetite are the most important symptoms to EG/EGE patients. The concepts relevant to patients with EG and EGE almost completely overlap, suggesting that it is appropriate to use a single PRO tool to measure symptoms in patients with these conditions.

The strengths and limitations of this study are common to all qualitative studies in this field. Qualitative methods allow for deep insight into the patient experience, including the salience and interrelationship between concepts. However, this methodology is limited by small sample sizes and potential biases in the recruitment of patients. For instance, since our sample included only patients from one country, speaking the same language, we may have phrased SAGED items in ways that not comprehensible to patients elsewhere. The current study sought to address these limitations in a variety of ways. Stopping the concept elicitation interviews only when we reached concept saturation (see the Methods section) gave us confidence that increasing the sample size and interviewing additional patients would not yield enough new information to change our conclusions. Following linguistic validation best practices, including back translation, cognitive interviewing, and developer review, supports the content validity of the tool in cross-cultural contexts.

## Conclusions

In conclusion, SAGED is a symptom assessment suitable for use in clinical studies in EG/EGE patients aged 12 and up. To form a comprehensive picture of clinical benefit in a clinical trial, SAGED should be supplemented with additional tools, such as PRO instruments for symptoms of secondary interest (e.g., fatigue) and HRQoL. The relationships between SAGED scores and other clinical outcomes should be explored. Evaluation of its measurement properties will further establish the appropriateness of using SAGED to measure symptom improvement in a clinical trial setting.

## Data Availability

The datasets generated and/or analyzed during the current study are not publicly available to protect patient privacy. Review copies of SAGED are available by request. A license agreement is required before use. Licenses are free of charge for academic and other non-commercial uses. Fees apply for commercial use. For inquiries about permission to use SAGED, please contact Calvin Ho (calvin.ho@astrazeneca.com).

## Footnotes

1 Pronounced /seɪdʒd/ like the wise ‘sage’ with a ‘d’ at the end.
